# The efficacy and neuroplasticity of Tuina combined with repetitive transcranial magnetic stimulation in the treatment of post-stroke depression: study protocol for a single-center, randomized, controlled trial

**DOI:** 10.3389/fneur.2025.1576620

**Published:** 2025-07-24

**Authors:** Zhao Ma, Zhengkun Gao, Xiao Chen, Yuqian Hu, Hang Gao, Jinying Gu, Haoxiang He, Hui Jin, Jiming Tao, Min Fang

**Affiliations:** Department of Rehabilitation, Shuguang Hospital, Shanghai University of Traditional Chinese Medicine, Shanghai, China

**Keywords:** Tuina, repetitive transcranial magnetic stimulation, post-stroke depression, functional magnetic resonance imaging, study protocol

## Abstract

**Introduction:**

Post-stroke depression (PSD), characterized by low mood and low interest, is the most common complication after stroke. The limitations of PSD drug therapy often require multidisciplinary combination therapy in clinical practice. Tuina therapy and repetitive transcranial magnetic stimulation (rTMS) have shown potential in modulating neural plasticity and improving depressive symptoms. However, the combined efficacy of these non-pharmacological therapies on PSD and their underlying mechanisms remain underexplored. This study aims to investigate the clinical effectiveness of massage combined with rTMS in treating PSD and explore its impact on brain functional networks and neuroendocrine mechanisms.

**Methods and analysis:**

This study is a non-inferiority randomized controlled trial (RCT). One hundred and twenty-eight participants with PSD will be randomly assigned (1:1) to the intervention group (Tuina + rTMS treatment) and the control group (cognitive behavior treatment). The primary efficacy outcome is the change from baseline to week 2 in Hamilton Depression Rating Scale (HAMD) and Functional Magnetic Resonance Imaging (fMRI). Secondary efficacy outcomes include other assessments of the Mini-Mental State Examination (MMSE), The modified Barthel index (MBI), The National Institutes of Health Stroke Scale(NIHSS), inflammatory factor (IL-6, IL – 17, TNF-*α*, IFN-*γ*, IL-4, IL-10), and Functional near-infrared spectroscopy (fNIRS). Efficacy and scale assessments will be conducted at 1 month and 3 months after the completion of treatment.

**Discussion:**

This trial will provide reliable evidence on the efficacy of Tuina combined with rTMS in treating PSD and explore its impact on brain functional networks and neuroendocrine mechanisms.

## Introduction

Post-stroke depression (PSD), characterized by low mood and low interest, is the most common complication after stroke. PSD can reduce the quality of life of patients, impair their cognitive function and life ability, and affect their process of rehabilitative treatment (1). Compared to other functional impairments after stroke, PSD is often overlooked (2). Relevant investigation results show that one third of stroke patients are affected by depression (3). PSD can occur at any time after stroke, with the highest prevalence during the first year and decreasing thereafter (4). It is worth noting that PSD is associated with poor outcome and increased mortality. The early diagnosis of PSD presents certain challenges. It relies on scale assessments, which are inherently subjective, and lacks objective diagnostic tools.

Although drug therapy is currently the primary clinical intervention for post-stroke depression, it exhibits numerous limitations (5). The efficacy of antidepressant drugs differs significantly among individuals, with some patients exhibiting poor response to conventional doses. Additionally, their adverse reactions can diminish patient compliance (6, 7). Drug therapy primarily focuses on symptom relief, with limited impact on promoting neuroplasticity and brain function recovery. Therefore, exploring safer complementary and alternative therapies is crucial for the treatment of PSD.

Tuina is a traditional non-drug therapy originating in China, which has demonstrated advantages in the treatment of depressive disorders. This therapy provides holistic regulation of the body, alleviating depressive symptoms while simultaneously addressing accompanying physical discomfort. It is essentially a tactile therapy that induces a pleasant sensory experience, which can alter the excitability and connectivity of brain regions involved in emotional regulation (8). There is research recommending massage as a B-level evidence for the treatment of depression disorders (9). A network meta-analysis has revealed that Tuina and other manual therapy are more effective than pharmacological interventions in reducing depressive symptoms in patients with depression of mild dementia (10). Additionally, multiple clinical studies have reported significant therapeutic efficacy of Tuina in the treatment of depression (11, 12).

Transcranial magnetic stimulation (TMS) is a non-invasive neuroregulation technique that has become a common treatment option for depression (13). Repetitive transcranial magnetic stimulation (rTMS) is a widely used form of TMS, characterized by the delivery of repeated magnetic pulses to specific cortical regions to modulate brain activity. By targeting depression-related brain regions, especially the dorsolateral prefrontal cortex (DLPFC) and associated emotional regulation networks, rTMS improves neural circuit function and promotes neuroplasticity (14). It has been recommended by the American Psychiatric Association (APA), as an effective treatment, particularly for treatment-resistant depression (15). A meta-analysis has demonstrated that rTMS can significantly improve outcomes in patients with PSD (16). Further research is essential to refine stimulation parameters, evaluate the long-term therapeutic effects, and explore the integration of rTMS with complementary approaches to enhance its efficacy.

Non-pharmacological therapies, with their reduced side effects and broader applicability, are gaining increasing attention in the treatment of PSD. Meta-analyses have confirmed their effectiveness, highlighting their potential as valuable therapeutic options (17–19). Among these, Tuina and rTMS have shown promise as separate interventions. Tuina therapy stimulates the spinal cord through peripheral interventions, promoting positive neuroplasticity in central emotional networks and restructuring neural circuits to treat PSD. Previous studies of our research group showed that Tuina improve PSD symptoms and enhance functional connectivity (FC) of hippocampus and thalamus (20). rTMS targets specific brain regions to enhance neural network function and plasticity. Tuina and rTMS act through distinct but potentially complementary mechanisms. While Tuina modulates brain function through bottom-up pathways initiated by peripheral stimulation, rTMS exerts top-down regulation by directly stimulating cortical targets. The combination of these two therapies may produce synergistic effects, contributing to improvements in emotional regulation, cognitive function, and limb function by integrating peripheral and central multi-level therapeutic mechanisms.

Despite advances in non-pharmacological treatments for PSD, the therapeutic potential of combining Tuina and rTMS remains unexplored. This study aims to evaluate the combined efficacy of Tuina and rTMS in improving emotional, cognitive, and functional outcomes in PSD patients. Using fMRI and fNIRS, this study investigates neural network remodeling and circuit reconstruction to elucidate the mechanisms underlying central regulation.

## Methods and analysis

### Study design

**Figure 1:**
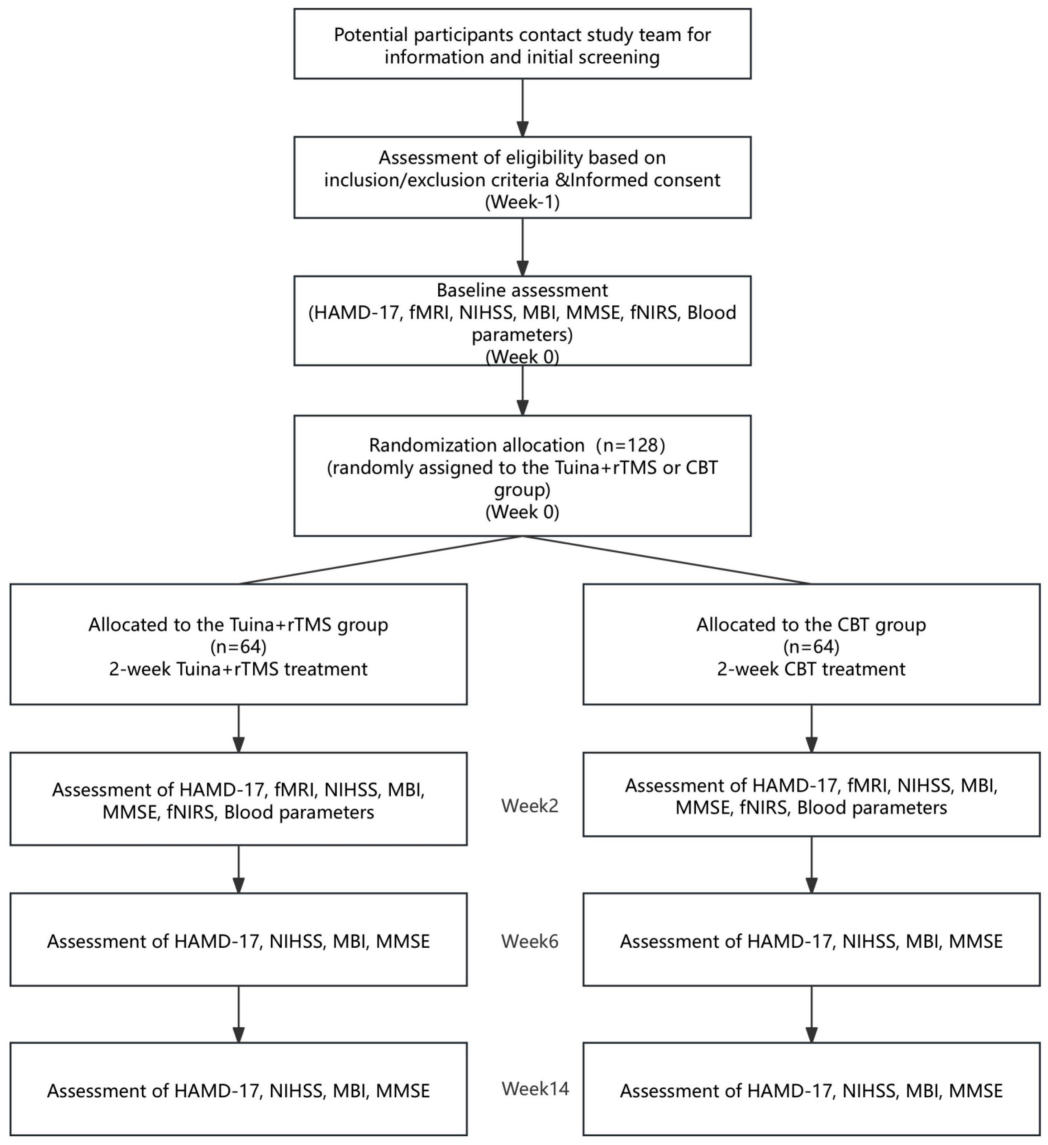
Participant flow chart.

This study is a single-center, parallel-design, analyst-blinded, randomized, controlled trial. A total of 128 PSD patients will be recruited and randomly assigned them in a 1:1 ratio to an intervention group and a control group. The intervention group will receive Tuina combined with rTMS, while the control group will undergo CBT. The study lasts 14 weeks and includes baseline assessments, a 2-week treatment phase, and a 3-month follow-up period. This study protocol has been developed in compliance with the SPIRIT 2013 guidelines for clinical trial reporting and adheres to the ethical principles outlined in the Declaration of Helsinki (version 2013). The study flowchart is presented in Figure 1, and the detailed study schedule is outlined in Table 1. The trial was registered at Chinese Clinical Trial Registry, https://www.chictr.org.cn/, identifier ChiCTR2400085310.

**Table 1:** Study schedule.

Study procedure	Period				
Time point	−1 week	0 week	2 week	6 week	14 week
Entry and administrative					
Eligibility screen	X				
Demographics	X				
Medical history	X				
Informed consent	X				
Randomization	X				
Interventions					
Tuina+rTMS		X	X		
CBT		X	X		
Outcome measures					
HAMD-17		X	X	X	X
fMRI		X	X		
NIHSS		X	X	X	X
MBI		X	X	X	X
MMSE		X	X	X	X
fNIRS		X	X		
Blood parameters		X	X		
Safety evaluations		X	X	X	X
Drug combinations		X	X	X	X
Patient compliance		X	X	X	X
Adverse events		X	X	X	X

### Participant recruitment

This study will be taken place in the inpatient unit of the Rehabilitation Department at Shuguang Hospital, affiliated with Shanghai University of Traditional Chinese Medicine. Recruitment announcements will be made through the hospital’s official social media account to maximize visibility and accessibility for potential participants. Patients will be recruited from the Rehabilitation Department, the Neurology Department, and local community centers. Additional recruitment efforts will include posters and the WeChat platform. Eligibility screening will take place at Shuguang Hospital, conducted by a designated recruitment physician.

### Inclusion criteria

Diagnostic criteria include:

- The diagnostic criteria of potential subjects refer to the Chinese Guidelines for Diagnosis and Treatment of Acute Ischemic Stroke 2018 (21), and based on The Chinese Expert Consensus on Clinical Practice for Post-Stroke Depression (22). At least three of the following symptoms must be present and persist for more than 1 week (with at least one of the symptoms from either item 1 or 2):

- Frequent occurrence of low mood.
- Loss of interest in daily activities.
- Significant reduction in energy or persistent fatigue without any obvious cause.
- Psychomotor retardation or agitation.
- Excessively low self-esteem, self-blame, or feelings of guilt reaching delusional severity.
- Lack of decisiveness, difficulty with associations, or a noticeable decline in cognitive abilities.
- Recurrent thoughts of death or suicidal ideation, or attempted suicide behavior.
- Insomnia, early awakening, or excessive sleep.
- Loss of appetite or significant weight loss.

- Age range: 45–80 years.
- Right-handed.
- Individuals with mild to moderate depression who score greater than 7, but less than or equal to 24 on the Hamilton Depression Rating Scale (HAMD-17).
- No consciousness impairment, no aphasia, cardiac function ≥ Grade 3, and stable vital signs.
- Patients must not have received antidepressant medications, Tuina therapy, or rTMS within the past 3 months.
- Complete screening and voluntarily sign the informed consent form (if the patient is unable to sign the informed consent due to physical impairments, the family member may sign on their behalf).

### Exclusion criteria

- Visual/hearing impairments, dementia (MMSE ≤ 20), cognitive dysfunction, or inability to complete assessments.
- Personal or family history of psychiatric disorders.
- Patients with severe suicidal tendencies.
- Acute (within 2 weeks) or progressive stroke, or severe systemic diseases (severe cardiovascular, hepatic, renal, respiratory, endocrine, or neurological diseases) with unstable vitals.
- Severe dermatological issues or local skin damage.
- Contraindications for TMS or MRI, such as pacemakers or metal implants.
- Use of antidepressant medication during the intervention period.

### Drop-out criteria

- Severe complications or worsening of the condition during the trial.
- Inability to tolerate Tuina or rTMS treatment.
- Withdrawal from the study or inability to complete the treatment.
- Development of other conditions deemed by the investigator to make continued participation in the clinical trial inappropriate.

### Randomization and blinding

Eligible participants will be randomly assigned to either the intervention group (Tuina + rTMS) or the control group (CBT) in a 1:1 ratio. A computer-generated randomization sequence will be prepared using the “proc plan” procedure in SAS 9.4 (SAS Institute Inc.), employing randomly permuted block sizes to ensure balanced group allocation. An independent researcher, uninvolved in recruitment, intervention, or data analysis, will generate the sequence. Each allocation will be concealed in a sequentially numbered, opaque, sealed envelope, securely stored to maintain allocation concealment. At the point of enrollment, the recruiting investigator will open the next envelope in sequence to determine group assignment. To minimize bias, outcome assessors, and statisticians will remain blinded to group allocation throughout the trial. Given the nature of the intervention, it is not feasible to blind PSD participants or treatment providers to group allocation. All participants will receive the same standard information about the study objectives and procedures at baseline without disclosing specific group assignments. Furthermore, both groups will receive interventions with identical frequency, duration, and therapist interaction time to control for nonspecific therapeutic effects. Statisticians, administrators, data collectors, and outcome assessors will be blinded during the study. And blinding will only be broken after data analysis is completed.

### Intervention

During the two-week study period, standard stroke treatments such as antiplatelet agents, antihypertensive drugs, antidiabetic medications, and lipid-lowering drugs will be permitted. The use of these medications will be meticulously documented for each participant.

### Intervention group

#### Tuina procedure

Tuina will be performed by an experienced, licensed therapists, who has more than 5 years of experience in clinical massage therapy. Participants lie in a prone or lateral position on therapy bed. The acupoints along the Governor Vessel (GV) are located, including Baihui (GV20), Dazhui (GV14), Zhiyang (GV9), Mingmen (GV4), and Changqiang (GV1). An ointment (1–3 g) is evenly applied to each acupoint. This ointment contains no active pharmacological ingredients and is used solely as a lubricant to facilitate manual techniques. The therapist applies thumb pressure and kneading techniques to each acupoint for 2 min. Subsequently, a three-finger massaging technique (index, middle, and ring fingers) is performed along the line connecting Dazhui (GV14) to Changqiang (GV1) for 10 min. Finally, palm-rubbing is used along the spine of the Governor Vessel from top to bottom for 30 s. Prior to treatment, therapists undergo 1 week of training using a TN-II massage technique measurement device. This ensures consistent application pressure of 0.5 kg and a frequency of 100 ± 10 strokes per minute during the procedures. Tuina therapy is conducted five times per week over 2 weeks, with each session lasting approximately 20 min.

#### rTMS intervention procedure

Patients are positioned comfortably in a seated or semi-reclined position on a therapy chair specifically designed for TMS treatment. The head is stabilized to minimize movement during the intervention. A figure-eight-shaped coil is used, placed tangentially to the scalp over the left dorsolateral prefrontal cortex (DLPFC), which has been identified as a key region associated with depression.

The stimulation parameters are set as follows:

*Frequency*: High-frequency stimulation (10 Hz) is commonly employed for left DLPFC.
*Intensity*: The intensity is typically set at 80% of the motor threshold (MT), individualized based on the patient’s resting MT, which is identified by observing the minimal intensity required to elicit a motor response in the contralateral abductor pollicis brevis muscle.
*Pulse train*: A single train consists of 20 pulses/sequences of stimulation followed by a 20-s rest period.
*Total pulses*: 60 sequences, 1,200 pulses per session.
*Frequency of sessions*: rTMS is administered once daily, five times per week, over a total of 2 weeks.

To ensure consistency and safety, all rTMS sessions are performed by certified clinicians under strict adherence to standardized protocols. Adverse events, such as mild headache or scalp discomfort, are closely monitored and documented during and after each session.

### Control group

Cognitive behavioral therapy is a widely recognized non-pharmacological intervention for PSD, focusing on modifying negative thought patterns and maladaptive behaviors that contribute to emotional emotional distress and impaired functioning. CBT training consists of a 3-h group-based health education seminar delivered at baseline to provide psychoeducation on stroke and emotional well-being, followed by a 2-week structured course comprising five sessions per week. Each CBT session lasts approximately 30 min and is conducted under the supervision of a qualified therapist with over 5 years of professional experience in psychotherapy. Interventions include:

- Disease education: Introduce the causes, clinical manifestations, and treatment methods of PSD formation to patients, enabling them to gain a basic understanding of the disease and guiding them to actively cooperate with treatment.
- Establishing trust: Proactively caring for patients, harmonizing doctor-patient relationships, enhancing doctor-patient trust, creating a warm, relaxed, and confident emotional environment for patients, and enhancing their sense of security.
- Identifying negative emotions: comprehensively understanding the patient’s emotions, mentality, exploring their negative factors and automatic thoughts.
- Changing Negative Cognition: Actively Identifying and Improving Potential Functional Impairment.

### Outcomes measures

#### General information collection

General information data include age, gender, education level, occupation, alcohol consumption and Smoking status.

#### Primary outcomes

The primary outcome measure for evaluating the severity of post-stroke depression is the 17-item Hamilton Depression Rating Scale (HAMD-17). This scale is a clinician-administered tool that quantitatively assesses depressive symptoms across multiple domains, including mood, anxiety, physical complaints, and psychomotor changes. HAMD-17 is particularly suitable for PSD as it captures somatic symptoms often present in stroke patients. A score >7 and ≤ 24 will be used to confirm mild to moderate depression, ensuring appropriate inclusion of participants in the study. Assessments will be conducted at baseline, midpoint (1 weeks), and at the end of the intervention (2 weeks) to evaluate changes in depressive symptoms over time. All evaluations will be performed by trained and blinded assessors to minimize measurement bias and maintain the integrity of the study.

In this study, rs-fMRI will serve as the second primary outcome measure to investigate neural mechanisms underlying post-stroke depression and its treatment effects. The imaging data were acquired using a United Imaging uMR780 3.0 T MRI system equipped with a 32-channel rigid head coil. Participants wore noise-canceling headphones, kept their eyes closed, remained awake, and minimized thoughts. The T1-weighted (T1W1) structural imaging parameters were as follows: TR = 7.2 ms, TE = 3.1 ms, flip angle = 10°, field of view (FOV) = 256 mm × 256 mm, slice thickness = 1 mm, and 192 slices. fMRI parameters included: TR = 2000 ms, TE = 30 ms, flip angle = 90°, FOV = 224 mm × 224 mm, matrix size = 64 × 64, and 33 slices. The voxel size was 3.5 mm × 3.5 mm × 3.5 mm, with 240 time points collected in each scan session, lasting 8 min per session.

Resting-state fMRI data were preprocessed using SPM12 software. The preprocessing steps included slice timing correction to account for temporal offsets in slice acquisition, realignment to correct for head motion, and coregistration of the functional images to the corresponding T1-weighted structural images. Segmentation of the T1 images was performed to generate normalization parameters, which were applied to transform the functional images into MNI space. Finally, spatial smoothing was conducted using a 6-mm FWHM Gaussian kernel to enhance the signal-to-noise ratio.

Regional Homogeneity (ReHo) is a data-driven method used in rs-fMRI to assess the synchronicity of spontaneous neural activity (23). It quantifies the temporal consistency of the BOLD signal within a given voxel and its 26 nearest neighbors, typically using Kendall’s coefficient of concordance (KCC). ReHo reflects the synchrony of local BOLD signal time series, rather than the signal intensity itself. This metric is widely employed to evaluate the functional integrity of local brain regions and their role in neural network dynamics, providing insights into regional brain activity and connectivity.

Amplitude of Low Frequency Fluctuations (ALFF) is widely used in fMRI to assess the strength of spontaneous brain activity in the resting state. By examining low-frequency fluctuations in the BOLD signal, typically within the 0.01–0.08 Hz range, ALFF provides insights into neural activity across different brain regions. A study has shown that severe depression patients exhibit significantly reduced ALFF values in the bilateral frontal poles after antidepressant treatment (24). There are also research findings, that increased ALFF values in the right inferior frontal gyrus and anterior insula have been associated with insomnia symptoms in depression, but not with anxiety or depressive symptom severity (25). These findings highlight ALFF’s utility in identifying regions with altered neural dynamics and their potential relationships to specific clinical features, offering valuable perspectives on the neural mechanisms underlying psychiatric disorders.

Functional Connectivity (FC) reflects the interdependence of neural signals across brain regions, providing insights into spontaneous neural activity and the coordination of brain networks during rest or task performance (26, 27). In fMRI, FC is assessed by measuring the temporal correlation of BOLD signal time series, either between predefined regions of interest (ROIs) or across the entire brain, to explore intrinsic neural networks and their dynamic interactions. Research indicates that FC between the anterior insula and the superior frontal regions is significantly higher in patients with PSD. This finding highlights altered neural network interactions that may underlie depressive symptoms in PSD.

Independent Component Analysis (ICA) will be performed using the Group ICA of fMRI Toolbox (GIFT, version 4.0, http://icatb.sourceforge.net) to decompose rs-fMRI data into independent components (ICs), allowing for the identification of functional networks within the brain. ICA is a data-driven technique that separates the observed fMRI signal into statistically independent sources, enabling the identification of distinct brain networks based on their temporal correlations.

#### Secondary outcomes

The Mini-Mental State Examination (MMSE) is widely used to assess the cognitive function in various clinical conditions, including post-stroke depression. This 30-point questionnaire evaluates domains such as orientation, attention, memory, language, and visuospatial abilities. A higher score indicates better cognitive function. In this study, MMSE will be used to evaluate cognitive status at baseline and after 12 weeks of intervention.

The modified Barthel index (MBI) is employed to evaluate the level of functional independence in activities of daily living (ADLs). The scale comprises 10 items, including feeding, bathing, grooming, dressing, and mobility. The scores range from 0 to 100, with higher scores reflecting greater independence. As PSD may negatively impact recovery in ADLs, the MBI serves as an essential secondary indicator of functional improvement.

The National Institutes of Health Stroke Scale (NIHSS) is a standardized tool used to quantify neurological deficits in stroke patients. It assesses multiple domains, including consciousness, motor strength, sensation, language, and visual fields. Scores range from 0 to 42, with higher scores indicating more severe deficits. Monitoring NIHSS scores provides insight into the interplay between PSD and the neurological recovery process.

Functional near-infrared spectroscopy (fNIRS) is conducted using two wavelengths of near-infrared light (690 nm and 830 nm) to measure oxygenated hemoglobin (oxy-Hb) based on the modified Beer–Lambert law. FNIRS assessments is performed at baseline, after 1 and 2 weeks of treatment. The study employed the Verbal Fluency Task (VFT) as a cognitive task to activate brain regions during fNIRS measurements, which is conducted on the same day as the tasks.

In this study, we selected IL-6, IL-4, IL-10, IL-17, TNF-*α*, interferon-*γ* (IFN-γ), adrenocorticotropic hormone (ACTH), and cortisol as key biomarkers to evaluate the interplay between inflammatory processes and hypothalamic–pituitary–adrenal (HPA) axis function in PSD. Pro-inflammatory cytokines such as IL-6, IL-17, TNF-α, and IFN-γ were measured to assess the level of systemic inflammation, which has been implicated in the pathophysiology of PSD. Anti-inflammatory cytokines, including IL-4 and IL-10, is evaluated to explore their potential regulatory effects. Additionally, ACTH and cortisol levels is used as indicators of HPA axis activation and stress response.

### Participant safety

When patients involved in this study, they will be required to understand the whole research plan, and sign the informed consent. In order to better prevention and treatment in this study may cause any damage, the researchers will detect any potential adverse events and truthfully recorded on the CRF table. In this study, adverse events is mainly related to treatment, including dizziness, headache, shortness of breath, palpitation. In the event of adverse events, will be in accordance with the contingency plans for processing. The investigator will first determine the severity of the adverse effects. Slight ae will by the doctor to treat. Serious ae report to the ethics committee will be made by the researchers.

### Quality control

This clinical study set up a quality inspector for the entire supervision. Test prior to the start of treatment of doctor training, unified specification operation, meet the requirements of technical specification rear can implement treatment. The relevant data collection will be completed between 8:00 a.m. and 16:00 a.m. A data monitoring committee, independent of the investigators, examined logic issues, ascertainment of values, processed abnormal safety indicators, missing values, compliance, standardization, completeness, and consistency throughout the study. The committee will be made by clinical physicians, statisticians, psychologist, and ethicist.

### Sample size calculation

The sample size for this study was calculated based on data from a previous clinical trial (20), in which the HAMD score was 9.90 ± 4.61 in the Tuina therapy group and 13.24 ± 4.36 in the conventional rehabilitation group. The combined standard deviation (*σ*) was 4.49. To detect a difference between group means (*Δ* = 2.5) with a statistical power of 80% (*β* = 0.2, corresponding to Zβ = 0.84) and a significance level of 0.05 (*α* = 0.05, corresponding to Zα/2 = 1.96), the required sample size per group was calculated using the following formula, with calculations performed in G*Power software to ensure adequate statistical power:


$$n=\frac{{\left(Z_{\alpha /2}+Z_{\beta }\right)}^{2}\cdot 2\sigma^{2}}{\Delta^{2}}$$

Thus, each group requires approximately 51 participants. Considering a 20% dropout rate, the adjusted sample size is 64. Therefore, a total of 64 participants per group will be required, resulting in a total sample size of 128 participants.

### Data collection and management

Two data managers who were not part of the study group and who were unaware of the group assignments were responsible for data entry and database establishment. All the related original data will be stored in Shuguang Hospital affiliated to Shanghai University of Traditional Chinese Medicine, and real-time uploaded to the Chinese clinical trial registry.

### Statistical analysis

#### Statistical analysis of scales and general information

All statistical analyses will be performed using SPSS version 26.0. Continuous variables will be presented as mean ± standard deviation (SD) and compared using independent t-tests or Mann–Whitney U tests based on data normality. Categorical variables will be expressed as frequencies and percentages and analyzed using chi-square or Fisher’s exact tests. Missing data will be handled using multiple imputation. To control for potential confounders, a multivariate regression analysis will be conducted. A two-tailed *p*-value < 0.05 will be considered statistically significant.

#### Statistical analysis of functional magnetic resonance imaging

In this study, fMRI data will undergo preprocessing using SPM12, including slice-timing correction, head motion correction, spatial normalization to the Montreal Neurological Institute (MNI) space, and spatial smoothing with a 6-mm Gaussian kernel. Functional brain networks will be constructed using both seed-based functional connectivity and ICA. For seed-based connectivity, regions of interest (ROIs) will be defined using the Automated Anatomical Labeling (AAL) atlas, and the mean BOLD signal from each ROI will be correlated to generate a connectivity matrix. Group ICA will be conducted using the GIFT software, decomposing the BOLD data into spatially independent components (ICs) that will be matched to canonical brain networks such as the default mode network (DMN), salience network (SN), and frontoparietal network (FPN). Additionally, graph-theoretical metrics, including degree centrality, clustering coefficient, and small-worldness, will be calculated to assess network efficiency and integration. Statistical analyses will involve comparing connectivity strength, network properties, and regional activations between groups using paired or independent t-tests, with corrections for multiple comparisons. Correlations between connectivity measures and clinical outcomes, such as HAMD scores, will be examined using Pearson or Spearman correlation coefficients. This comprehensive approach ensures robust exploration of functional brain networks and their clinical relevance.

#### Statistical analysis of near infrared brain functional imaging

To evaluate statistical differences between the two groups, *t*-tests are conducted using MATLAB 2017 (MathWorks). Specifically, independent sample *t*-tests are performed to compare the mean activation levels between the two groups, while paired *t*-tests are used to assess activation changes within the PSD group before and after treatment. All data are presented as mean ± SD. Additionally, to validate the results of functional connectivity analyses, both independent sample and paired *t*-tests are applied.

#### Statistical analysis of functional magnetic resonance imaging

Image data analysis is performed using Network Based Statistics (NBS) V1.2 statistical software based on MATLAB platform to construct local brain functional networks for two groups of subjects. Brain network features within each group are constructed through single sample *t*-test. The network between the observation group and the control group before and after treatment is compared using a two independent sample *t*-test; Multiple comparisons between local brain functional networks are corrected for false discovery rate (FDR). The difference is considered statistically significant with *p* < 0.05. The results of brain function data are presented using BrainNet Viewer 1.6 software. The significant brain regions detected before and after treatment are compared between the control group and the observation group. SPSS 25.0 software is used to perform correlation analysis with clinical scale scores. Pearson correlation is selected for normal distribution, while Spearman correlation is selected for non-normally distributed data. *p* < 0.05 is considered statistically significant. The results of the relevant analysis data are presented using the statistical parameter graph software Origin 2021.

## Discussion

PSD is a common and disabling condition that significantly impacts the rehabilitation outcomes of stroke survivors. It is characterized by a complex interplay of biological, psychological, and social factors, making its management challenging. Although there have been advances in drug and psychological treatments, many PSD patients still do not respond well to current therapies, showing the need to explore alternative and complementary methods. As a traditional Chinese medicine therapy, Tuina can effectively alleviate depression and improve cognitive function in PSD patients by activating the hippocampus and regulating brain functional connections (20). In recent years, rTMS, as a non-invasive brain stimulation technique, has also shown promise in modulating cortical excitability and improving mood in depressive disorders (28). This approach combines the holistic benefits of TCM with the precise neurophysiological effects of TMS, offering an innovative treatment strategy.

The pathogenesis of PSD is relatively complex. Structural magnetic resonance imaging and fMRI can reflect changes in brain morphology and function, and can be used for visualizing unknown brain lesions in psychiatric and neurological disorders (29). Dysfunction in functional brain networks may play a critical role in the development of PSD (30). Studies have shown significant reorganization of functional brain networks in PSD patients (31). First introduced by Raichle (32), the concept of the DMN identifies the precuneus and posterior cingulate cortex as key nodes within this network. These regions exhibit significantly higher metabolic activity during the resting state compared to other brain areas (33). Additionally, disruptions in the functional connectivity of the default mode network, which are closely associated with emotional regulation, have been linked to the severity of PSD symptoms (34). Research has demonstrated that reduced FC in the precuneus, posterior cingulate cortex, and angular gyrus of the DMN significantly correlates with anxiety and depression scores in patients (35). Moreover, the pathological changes in PSD are also closely related to the limbic system-cortico-striato-pallido-thalamo-cortical circuit (36), which plays a fundamental role in emotional regulation and neuroplasticity. Evidence suggests that massage therapy can activate the hippocampus and insula, promoting functional remodeling of the limbic system, thereby improving emotional and cognitive functions (37, 38). fNIRS is characterized by its non-invasive nature, portability, and high safety profile, making it well-suited for real-time monitoring of cortical activity in clinical and research settings. By monitoring oxygen metabolism changes in specific cortical regions, it can identify functional abnormalities in key brain areas related to emotional regulation and cognitive function, such as the prefrontal cortex and limbic system, and evaluate the modulatory effects of interventions on brain functional networks (39). Studies have shown that changes in internal and external environments can activate the HPA axis, and dysregulation of the HPA axis is closely associated with the pathogenesis of PSD (40). Approximately 40% of stroke patients exhibit HPA axis hyperactivity, characterized by elevated glucocorticoid levels and hypercortisolism (41). This study uses advanced neuroimaging and biochemical methods to explore the neural mechanisms of PSD and assess the impact of intervention. Investigating the characteristics and remodeling patterns of functional brain networks following Tuina combined with rTMS intervention can help identify activated brain regions and associated neural circuits.

The significance of this study lies in its potential to provide robust evidence on the combined effects of Tuina and TMS for treating PSD. By evaluating both clinical outcomes, such as depressive symptoms and functional recovery, and neurophysiological mechanisms through advanced imaging and biomarker analyses, this research aims to bridge the gap between traditional therapies and modern neuroscience. It also seeks to establish a standardized protocol for the combined intervention, contributing to the evidence base for integrative medicine in stroke rehabilitation.

Our clinical study also has some limitations. First, as the intervention is a non-pharmacological treatment, implementing a double-blind design is challenging, which may introduce potential bias. To address this limitation, we ensured that outcome assessors and data analysts remain blinded throughout the trial. In addition, participants in both groups receive consistent instructions and equal levels of therapist contact, with matched treatment frequency and duration. These measures are intended to reduce expectation-related bias and help account for nonspecific intervention effects. Second, the sample size, although the sample size is relatively modest and may limit the generalizability of the findings to broader populations, it was carefully calculated based on effect size estimates from previous studies and includes a buffer for potential dropout. Participants were recruited from multiple clinical departments within a tertiary hospital and nearby community health centers, which we believe captures a diverse and representative PSD population. These design choices aim to balance statistical rigor with practical feasibility, thereby enhancing both the internal validity and real-world relevance of the study. Moreover, variations in individual patient responsiveness to TMS or massage due to underlying neurobiological or psychological factors may also affect the generalizability of the results. Patients’ expectations regarding the effectiveness of treatment should also be considered in future research.

## References

[1] Zhou H Wei YJ Xie GY. Research progress on post-stroke depression. Exp Neurol. (2024) 373:114660. doi: 10.1016/j.expneurol.2023.114660, PMID: 38141804

[2] Hackett ML Anderson CS House A Halteh C. Interventions for preventing depression after stroke. Cochrane Database Syst Rev. (2008) 3:CD003689. doi: 10.1002/14651858.CD003689.pub3 18646094

[3] Sun S Li Z Xiao Q Tan S Hu B Jin H. An updated review on prediction and preventive treatment of post-stroke depression. Expert Rev Neurother. (2023) 23:721–39. doi: 10.1080/14737175.2023.2234081, PMID: 37427452

[4] Jeong S Chokkalla AK Davis CK Vemuganti R. Post-stroke depression: epigenetic and epitranscriptomic modifications and their interplay with gut microbiota. Mol Psychiatry. (2023) 28:4044–55. doi: 10.1038/s41380-023-02099-8, PMID: 37188778 PMC10646155

[5] Hackett ML Köhler S O'Brien JT Mead GE. Neuropsychiatric outcomes of stroke. Lancet Neurol. (2014) 13:525–34. doi: 10.1016/s1474-4422(14)70016-x, PMID: 24685278

[6] Frank D Gruenbaum BF Zlotnik A Semyonov M Frenkel A Boyko M. Pathophysiology and current drug treatments for post-stroke depression: a review. Int J Mol Sci. (2022) 23. doi: 10.3390/ijms232315114, PMID: 36499434 PMC9738261

[7] Masuccio FG Grange E Di Giovanni R Rolla M Solaro CM. Post-stroke depression in older adults: an overview. Drugs Aging. (2024) 41:303–18. doi: 10.1007/s40266-024-01104-1, PMID: 38396311

[8] Eggart M Queri S Muller-Oerlinghausen B. Are the antidepressive effects of massage therapy mediated by restoration of impaired interoceptive functioning? A novel hypothetical mechanism. Med Hypotheses. (2019) 128:28–32. doi: 10.1016/j.mehy.2019.05.004, PMID: 31203905

[9] Greenlee H DuPont-Reyes MJ Balneaves LG Carlson LE Cohen MR Deng G . Clinical practice guidelines on the evidence-based use of integrative therapies during and after breast cancer treatment. CA Cancer J Clin. (2017) 67:194–232. doi: 10.3322/caac.21397, PMID: 28436999 PMC5892208

[10] Watt JA Goodarzi Z Veroniki AA Nincic V Khan PA Ghassemi M . Comparative efficacy of interventions for reducing symptoms of depression in people with dementia: systematic review and network meta-analysis. BMJ. (2021) 372:n532. doi: 10.1136/bmj.n532, PMID: 33762262 PMC7988455

[11] Chen Y Sun XY Qian C Zhang XX Zhou YJ Zhang HY . Therapeutic effect of manual massage on early postpartum rectus abdominis separation and postpartum depression. World J Psychiatry. (2024) 14:678–85. doi: 10.5498/wjp.v14.i5.678, PMID: 38808091 PMC11129154

[12] Lyman GH Greenlee H Bohlke K Bao T DeMichele AM Deng GE . Integrative therapies during and after breast cancer treatment: ASCO endorsement of the SIO clinical practice guideline. J Clin Oncol. (2018) 36:2647–55. doi: 10.1200/jco.2018.79.2721, PMID: 29889605 PMC13123314

[13] Cappon D den Boer T Jordan C Yu W Metzger E Pascual-Leone A. Transcranial magnetic stimulation (TMS) for geriatric depression. Ageing Res Rev. (2022) 74:101531. doi: 10.1016/j.arr.2021.101531, PMID: 34839043 PMC8996329

[14] Lefaucheur JP Aleman A Baeken C Benninger DH Brunelin J Di Lazzaro V . Evidence-based guidelines on the therapeutic use of repetitive transcranial magnetic stimulation (rTMS): an update (2014-2018). Clin Neurophysiol. (2020) 131:474–528. doi: 10.1016/j.clinph.2019.11.002, PMID: 31901449

[15] Perera T George MS Grammer G Janicak PG Pascual-Leone A Wirecki TS. The clinical TMS Society consensus review and treatment recommendations for TMS therapy for major depressive disorder. Brain Stimul. (2016) 9:336–46. doi: 10.1016/j.brs.2016.03.010, PMID: 27090022 PMC5612370

[16] Gao W Xue F Yu B Yu S Zhang W Huang H. Repetitive transcranial magnetic stimulation for post-stroke depression: an overview of systematic reviews. Front Neurol. (2023) 14:930558. doi: 10.3389/fneur.2023.930558, PMID: 37006488 PMC10061017

[17] Yanyu S Ying L Kexin L Jin W. Non-invasive brain stimulation for treating post-stroke depression: a network meta-analysis. Int J Geriatr Psychiatry. (2023) 38:e5941. doi: 10.1002/gps.5941, PMID: 37283525

[18] Yi Y Zhao W Lv S Zhang G Rong Y Wang X . Effectiveness of non-pharmacological therapies for treating post-stroke depression: a systematic review and network meta-analysis. Gen Hosp Psychiatry. (2024) 90:99–107. doi: 10.1016/j.genhosppsych.2024.07.011, PMID: 39084147

[19] Xiao K Li X Hu W Li X. Acupuncture combined with repetitive transcranial magnetic stimulation for the treatment of post-stroke depression: a systematic evaluation and meta-analysis based on a randomised controlled trial. Front Neurol. (2024) 15:1360437. doi: 10.3389/fneur.2024.1360437, PMID: 38817548 PMC11137222

[20] Tao J Zhang S Kong L Zhu Q Yao C Guo Q . Effectiveness and functional magnetic resonance imaging outcomes of Tuina therapy in patients with post-stroke depression: a randomized controlled trial. Front Psych. (2022) 13:923721. doi: 10.3389/fpsyt.2022.923721, PMID: 35845459 PMC9281445

[21] Chinese Society of Neurology, Chinese Stroke Society. Chinese guidelines for diagnosis and treatment of acute ischemic stroke 2018 (2018). doi: 10.3760/cma.j.issn.1006-7876.2018.09.004,

[22] Wang SZX Zhu C. Chinese expert consensus on clinical practice for post-stroke depression. Chin J Stroke. (2016) 11:685–93.

[23] Chen VC Chou YS Tsai YH Huang YC McIntyre RS Weng JC. Resting-state functional connectivity and brain network abnormalities in depressive patients with suicidal ideation. Brain Topogr. (2021) 34:234–44. doi: 10.1007/s10548-020-00817-x, PMID: 33420533

[24] Chen F Lv X Fang J Yu S Sui J Fan L . The effect of body-mind relaxation meditation induction on major depressive disorder: a resting-state fMRI study. J Affect Disord. (2015) 183:75–82. doi: 10.1016/j.jad.2015.04.030, PMID: 26001666

[25] Liu CH Guo J Lu SL Tang LR Fan J Wang CY . Increased salience network activity in patients with insomnia complaints in major depressive disorder. Front Psych. (2018) 9:93. doi: 10.3389/fpsyt.2018.00093, PMID: 29615938 PMC5869937

[26] Suarez LE Markello RD Betzel RF Misic B. Linking structure and function in macroscale brain networks. Trends Cogn Sci. (2020) 24:302–15. doi: 10.1016/j.tics.2020.01.008, PMID: 32160567

[27] Maggioni E Tana MG Arrigoni F Zucca C Bianchi AM. Constructing fMRI connectivity networks: a whole brain functional parcellation method for node definition. J Neurosci Methods. (2014) 228:86–99. doi: 10.1016/j.jneumeth.2014.03.004, PMID: 24675050

[28] Zhu Z Zhu HX Jing SW Li XZ Yang XY Luo TN . Effect of transcranial magnetic stimulation in combination with citalopram on patients with post-stroke depression. Front Hum Neurosci. (2022) 16:962231. doi: 10.3389/fnhum.2022.962231, PMID: 36277050 PMC9585658

[29] Sarkar A Sarmah D Datta A Kaur H Jagtap P Raut S . Post-stroke depression: chaos to exposition. Brain Res Bull. (2021) 168:74–88. doi: 10.1016/j.brainresbull.2020.12.012, PMID: 33359639

[30] Zhang P Xu Q Dai J Wang J Zhang N Luo Y. Dysfunction of affective network in post ischemic stroke depression: a resting-state functional magnetic resonance imaging study. Biomed Res Int. (2014) 2014:846830. doi: 10.1155/2014/846830, PMID: 24963485 PMC4053214

[31] Dai L Zhou H Xu X Zuo Z. Brain structural and functional changes in patients with major depressive disorder: a literature review. PeerJ. (2019) 7:e8170. doi: 10.7717/peerj.8170, PMID: 31803543 PMC6886485

[32] Raichle ME Snyder AZ. A default mode of brain function: a brief history of an evolving idea. NeuroImage. (2007) 37:1083–90. doi: 10.1016/j.neuroimage.2007.02.041, PMID: 17719799

[33] Gusnard DA Raichle ME Raichle ME. Searching for a baseline: functional imaging and the resting human brain. Nat Rev Neurosci. (2001) 2:685–94. doi: 10.1038/35094500, PMID: 11584306

[34] Lassalle-Lagadec S Sibon I Dilharreguy B Renou P Fleury O Allard M. Subacute default mode network dysfunction in the prediction of post-stroke depression severity. Radiology. (2012) 264:218–24. doi: 10.1148/radiol.12111718, PMID: 22668562

[35] Coutinho JF Fernandesl SV Soares JM Maia L Goncalves OF Sampaio A. Default mode network dissociation in depressive and anxiety states. Brain Imaging Behav. (2016) 10:147–57. doi: 10.1007/s11682-015-9375-7, PMID: 25804311

[36] Terroni L Amaro E Iosifescu DV Tinone G Sato JR Leite CC . Stroke lesion in cortical neural circuits and post-stroke incidence of major depressive episode: a 4-month prospective study. World J Biol Psychiatry. (2011) 12:539–48. doi: 10.3109/15622975.2011.562242, PMID: 21486107 PMC3279135

[37] Xiaohao HSS. Application of combined acupuncture and massage therapy in BOLD-fMRI studies for hemiplegic patients. Chin J Integr Med Cardio-Cerebrovasc Dis. (2020) 18:3950–3.

[38] Li HZW Siwen L Xiongfei L Wei F Fei M G W. Application progress of fMRI technology in massage scientific research Tianjin Chinese. Tianjin J Tradit Chin Med. (2019) 36:309–12.

[39] Kassubek J. The application of neuroimaging to healthy and diseased brains: present and future. Front Neurol. (2017) 8:61. doi: 10.3389/fneur.2017.00061, PMID: 28286495 PMC5323372

[40] Lichlyter DA Krumm ZA Golde TA Dore S. Role of CRF and the hypothalamic-pituitary-adrenal axis in stroke: revisiting temporal considerations and targeting a new generation of therapeutics. FEBS J. (2023) 290:1986–2010. doi: 10.1111/febs.16380, PMID: 35108458

[41] Villa RF Ferrari F Moretti A. Post-stroke depression: mechanisms and pharmacological treatment. Pharmacol Ther. (2018) 184:131–44. doi: 10.1016/j.pharmthera.2017.11.005, PMID: 29128343

